# An Integrated Biochemical, Proteomics, and Metabolomics Approach for Supporting Medicinal Value of *Panax ginseng* Fruits

**DOI:** 10.3389/fpls.2016.00994

**Published:** 2016-07-04

**Authors:** So W. Kim, Ravi Gupta, Seo H. Lee, Cheol W. Min, Ganesh K. Agrawal, Randeep Rakwal, Jong B. Kim, Ick H. Jo, Soo-Yun Park, Jae K. Kim, Young-Chang Kim, Kyong H. Bang, Sun T. Kim

**Affiliations:** ^1^Department of Plant Bioscience, Life and Industry Convergence Research Institute, Pusan National University, MiryangSouth Korea; ^2^Research Laboratory for Biotechnology and Biochemistry, KathmanduNepal; ^3^Global Research Arch for Developing Education Academy Private Limited, BirgunjNepal; ^4^Faculty of Health and Sport Sciences and Tsukuba International Academy for Sport Studies, University of Tsukuba, IbarakiJapan; ^5^Global Research Center for Innovative Life Science, Peptide Drug Innovation, School of Pharmacy and Pharmaceutical Sciences, Hoshi University, TokyoJapan; ^6^Department of Biotechnology, College of Biomedical and Health Sciences, Konkuk University, Choong-JuSouth Korea; ^7^Department of Herbal Crop Research, Rural Development Administration, EumseongSouth Korea; ^8^National Academy of Agricultural Science, Rural Development Administration, Jeollabuk-doSouth Korea; ^9^Division of Life Sciences, Incheon National University, IncheonSouth Korea

**Keywords:** proteome, metabolome, 2D reference map, antioxidants, fruits, ginseng

## Abstract

*Panax ginseng* roots are well known for their medicinal properties and have been used in Korean and Chinese traditional medicines for 1000s of years. However, the medicinal value of *P. ginseng* fruits remain poorly characterized. In this study, we used an integrated biochemical, proteomics, and metabolomics approach to look into the medicinal properties of ginseng fruits. DPPH (1,1-diphenyl-2-picrylhydrazyl) and ABTS [2,2′-azino-bis (3-ethylbenzothiazoline-6-sulphonic acid)] assays showed higher antioxidant activities in ginseng fruits than leaves or roots. Two-dimensional gel electrophoresis (2-DE) profiling of ginseng fruit proteins (cv. Cheongsun) showed more than 400 spots wherein a total of 81 protein spots were identified by mass spectrometry using NCBInr, UniRef, and an in-house developed RNAseq (59,251 protein sequences)-based databases. Gene ontology analysis showed that most of the identified proteins were related to the hydrolase (18%), oxidoreductase (16%), and ATP binding (15%) activities. Further, a comparative proteome analysis of four cultivars of ginseng fruits (cvs. Yunpoong, Gumpoong, Chunpoong, and Cheongsun) led to the identification of 22 differentially modulated protein spots. Using gas chromatography-time of flight mass spectrometry (GC-TOF MS), 66 metabolites including amino acids, sugars, organic acids, phenolic acids, phytosterols, tocopherols, and policosanols were identified and quantified. Some of these are well known medicinal compounds and were not previously identified in ginseng. Interestingly, the concentration of almost all metabolites was higher in the Chunpoong and Gumpoong cultivars. Parallel comparison of the four cultivars also revealed higher amounts of the medicinal metabolites in Chunpoong and Gumpoong cultivars. Taken together, our results demonstrate that ginseng fruits are a rich source of medicinal compounds with potential beneficial health effects.

## Introduction

Ginseng (*Panax ginseng* C. A. Meyer) belongs to the genus *Panax* of the Araliaceae family. “*Panax*” term is derived from the Greek word “panacea,” which is a combination of two words “Pan-,” meaning all, and “axos,” meaning cure, due wide use of ginseng as a medicine (Attele et al., 1999). Ginseng root has long been used as a traditional herbal medicinal crop due to its diverse effects on brain function improvement, anti-tumor activity, immune system function enhancement, and anti-aging effects (Vogler et al., 1999; Jia et al., 2009). Because of the medicinal properties, ginseng research was mainly focused on the isolation and characterization of its medicinal ingredients such as saponins and phenolic compounds from the roots (Sun, 2011; Chung et al., 2016). Reports suggest that ginseng fruits also exhibit medicinal properties, where the saponin content of fruits is four times higher than that of roots (Cho et al., 2006; Wang et al., 2006; Ko et al., 2008; Kim et al., 2009; Lee et al., 2010). However, information regarding the other potential bioactive/medicinal metabolites present in ginseng fruits is still scarce and needs to be investigated.

As of today, very few reports have been published on the proteomic and metabolomic analyses of different tissues of ginseng. Comparative proteomic and metabolomic analyses have been performed on many crop and medicinal plants to examine differentially expressed proteins or metabolites under adverse conditions and in different cultivars (Purkayastha et al., 2014; Gupta et al., 2015b; Min et al., 2015; Ma et al., 2016). Metabolome studies have been performed on ginseng to assess the effects of colored light-emitting diode lighting (Park et al., 2013), cultivation ages (Kim et al., 2011) on adventitious roots, and environmental conditions of main roots (Kim et al., 2010). Attempts have also been made to analyze the ginseng stem proteins (Sun et al., 2011), hairy root proteins (Kim et al., 2003), root storage proteins (Kim et al., 2004), growth-associated proteins (Ma et al., 2013), and stress-responsive proteins (Kim et al., 2014). However, the ginseng fruit proteome and metabolome have not yet been investigated, and such research could be useful to gain a new understanding on the medicinal properties contained within these fruits.

Therefore, here we employed, for the very first time to the best of our knowledge, a combination of biochemical, proteomics and metabolomics approach to investigate the medicinal properties of ginseng fruits through unraveling of these molecular components. Moreover, a comparative proteomics analysis was also carried out on ginseng fruits obtained from four different cultivars to identify the cultivar-specific proteins.

## Materials and Methods

### Plant Materials

In this study, four *P. ginseng* cultivars were used. These are: (i) Yunpoong with violet stem and red fruits, (ii) Gumpoong with green stem and yellow fruits, (iii) Chunpoong with majorly green stem and orange fruits, and (iv) Cheongsun with green stem and red fruits. Each cultivar grown in the greenhouse (average temperature 22.5 ± 2.5°C, humidity 50 ± 10%) at the National Institute of Horticultural and Herbal Science, Rural Development Administration (RDA) at Eumseong-gun, South Korea (36°N, 127°E). Four years into its growing season, in the month of July, the roots, leaves, and fruits were harvested, powdered using liquid nitrogen and stored in a deep freezer (-80°C) until further processing.

### DPPH and ABTS Radical Scavenging Activities Assay

Fine powder (1 g) of each ginseng tissues was mixed with 10 ml of 100% methanol and shaken at 180 rpm for 24 h. The mixture was filtered using a syringe filter (0.20 μm, Sartorius Stedim Biotech). The DPPH (1,1-diphenyl-2-picrylhydrazyl) assay was performed as described previously (Brandwilliams et al., 1995). Briefly, 40 mM DPPH solution was prepared in 100% methanol and its absorbance was adjusted to 0.93 ± 0.03 at 525 nm. DPPH solution (180 μl) was mixed with either 20 μl 10% ascorbic acid (positive control) or test samples and incubated for 30 min in the dark. After 30 min, the reduction of absorbance was monitored at 525 nm. Absorbance of each sample was measured in triplicate. DPPH radical scavenging capacity was calculated using the following equation.

$$DPPHscavengingeffect\left(\%\right)\,=\;\left[(A_{1}-A_{2})/A_{0}\right]\,\times \;100,$$

where A_0_ and A_1_ correspond to the absorbances of the radical in the absence and presence of antioxidant at 525 nm, respectively. A_2_ represents absorbance of the sample mixed with methanol at 525 nm.

ABTS [2,2′-azino-bis(3-ethylbenzothiazoline-6-sulphonic acid)] assay was performed as described previously (Van den Berg et al., 1999). ABTS solution was prepared using 10 ml of 7.4 mM ABTS mixed with 10 ml of 2.6 mM potassium persulfate and incubated for 24 h in the dark. This solution was further diluted using 100% methanol until its absorbance was 0.73 ± 0.03 at 732 nm. The overall procedure of ABTS assay was similar to the DPPH assay except for the incubation time and wavelength. In the case of ABTS assay, a 5 min incubation time was required, and the absorbance was measured at 732 nm.

The IC_50_ value, which indicates half maximal inhibitory concentration, was calculated by linear regression analysis. Results are given as means ± standard deviation of three measurements. Statistical analysis was performed using one way-ANOVA followed by Tukey’s Kramer multiple comparisons test; *p* < 0.05 was considered to be significant.

### Protein Extraction

Total proteins from fruits of four ginseng cultivars were isolated as described previously (Kim U.G. et al., 2012; Gupta et al., 2015a). The prepared fine powder (1 g) of ginseng fruits was homogenized in 10 ml of ice-cold Tris-Mg/NP-40 extraction buffer [0.5 M Tris-HCl (pH 8.3), 2% (v/v) NP-40, 20 mM MgCl_2_, and 2% (v/v) β–mercaptoethanol] and centrifuged at 12,000 × *g* for 10 min at 4°C. To the collected supernatant, four volumes of 12.5% (w/v) TCA/acetone was added, mixed thoroughly, and incubated at -20°C for 1 h. After centrifugation at 12,000 × *g* for 5 min at 4°C, the pellet was washed two times with cold 80% (v/v) acetone in distilled water containing 0.07% β–mercaptoethanol. Finally, the pellet was suspended in 80% acetone and stored at -20°C until further use.

### 2-DE and Image Analysis

Two-dimensional electrophoresis (2-DE) and gel-image analysis were performed as reported previously (Kim U.G. et al., 2012). Briefly, total proteins (600 μg) were loaded onto 24 cm IPG strips (immobilized pH 4-7; GE Healthcare, Waukesha, WI, USA) by rehydration loading overnight. Iso-electric focusing was performed at 50 V for 4 h, 100 V for 1 h, 500 V for 1 h, 1000 V for 1 h, 2000 V for 1 h, 4000 V for 2 h, 8000 V for 5 h, 8000 V for 9 h, and 50 V for 6 h using the IPGphor II platform (GE Healthcare, Waukesha, WI, USA). Each focused strip was equilibrated in 5 ml of equilibration buffer containing 6 M urea, 30% (v/v) glycerol, 2% (w/v) SDS, 50 mM Tris-HCl (pH 6.8), 0.1 mg/ml of bromophenol blue, and 100 mM DTT as a first step, and 55 mM iodoacetamide as the second step. Second dimensional separation of proteins was carried out on 14% SDS-PAGE at 2 W per gel, 500 V, and 300 mA for 30 min, followed by 16 W per gel, 700 V, and 300 mA. Gels were stained with colloidal CBB (Coomassie Brilliant Blue) G-250 staining solution [34% (v/v) methanol, 17% (w/v) ammonium sulfate, 3% (v/v) phosphoric acid, and 0.1% (w/v) CBB G-250] and destained twice with 30% (v/v) methanol distilled water. The stained gels were scanned for image analysis using a transmissive scanner (PowerLook 1120, UMAX, Dallas, TX, USA) with a 32 bit pixel depth and 300 dpi resolution. Protein spots on 2D gels were detected and processed using ImageMaster 2D Platinum software 6.0 (GE Healthcare, Waukesha, WI, USA). Percentage volume of each spot was determined from three biological replicates. Statistical analysis was performed using one way-ANOVA followed by Turkey’s multiple comparisons test; *p* < 0.05 was considered to be significant.

### Protein Analysis by MALDI-TOF/TOF MS and LC-MS/MS

The selected protein spots were excised from the 2-D gels and subjected to in-gel digestion with trypsin as described previously (Kim et al., 2008). Matrix Assisted Laser Desorption Ionization-Time Of Flight/Time Of Flight Mass Spectrometry (MALDI-TOF/TOF MS) analysis was carried out using an ABI 4800 Plus TOF-TOF MS (Applied Biosystems, Framingham, MA, USA) as described previously (Kwon et al., 2010). The 10 most and least intense ions per MALDI spot, with signal/noise ratios >25, were selected for subsequent MS/MS analysis in 1 kV mode and 800–1,000 consecutive laser shots. During MS/MS analysis, air was used as the collision gas. Data were subjected to a Mass Standard Kit for the 4700 Proteomics Analyzer (calibration Mixture 1). MS/MS spectra were searched against the NCBInr/UniRef/RNAseq-based (srx1791675) in-house developed databases, which contained protein information (33,815,671 sequences; 11,795,550,776 residues/30,938,908 sequences; 11,077,485,688 residues/59,251 sequences; 1,138,902 residues) by ProteinPilot v.3.0 software (AB Sciex, Framingham, MA, USA) using MASCOT (ver. 2.3.0, Matrix Science, London, UK) as the database search engine, with peptides and a fragment ion mass tolerance set to 50 ppm. Carbamidomethylation of cysteines and oxidation of methionine were allowed during the peptide search. One missing trypsin cleavage was allowed. Peptide mass tolerance and fragment mass tolerance of the selected proteins were set to 50 ppm. High confidence identifications showed statistically significant search scores (greater than 95% confidence, equivalent to MASCOT expect value *p* < 0.05), which were consistent with each protein’s experimental *p*I and MW, and accounted for the majority of ions present in the mass spectra.

Liquid Chromatography-Mass Spectrometry/Mass Spectro metry (LC-MS/MS) experiment was performed using an integrated system consisting of high-performance liquid chromatography (HPLC) interfaced to linear trap quadropole-orbitrap (LTQ-Orbitrap, Thermo Fisher Scientific, USA), equipped with a nano-electrospray ionization source, and fitted with a fused silica emitter tip (New Objective, Woburn, MA, USA). Peptidases were reconstituted in Solvent A [water/ACN (98:2, v/v), and 0.1% formic acid], and then injected into the liquid chromatography-nano ElectroSpray ionization-mass spectrometry/mass spectrometry (LC-nESI-MS/MS system). Samples were first trapped on a Zorbax 300SB-C18 trap column (5 μm, 0.3 mm × 5 mm, Agilent Technologies, part number 5065–9913), washed for 10 min with 98% solvent A, 2% solvent B [Water/ACN (2:98, v/v), and 0.1% formic acid] at a flow rate of 5 μl/min, and then separated on a Zorbax 300SB-C18 capillary column (3.5 μm, 150 × 0.075 mm, Agilent Technologies, part number 5065–9911) at a flow rate of 300 nl/min. The LC gradient was run from 2 to 35% solvent B over 60 min, and from 35 to 90% over 10 min, followed by 90% solvent B for 5 min, and finally 5% solvent B for 15 min. Resulting peptides were electrosprayed through a coated silica tip (FS360-20-10-N20-C12, PicoTip emitter, New Objective) at an ion spray voltage of 2,000 eV. The raw data from LTQ-Orbitrap was processed using Proteome Discoverer 1.3 software (Thermo Fisher Scientific, USA).

### Metabolite Analysis by GC-TOF MS

Hydrophilic metabolites were extracted from the powdered fruit sample (10 mg) by addition of 1 ml of 2.5:1:1 (v/v/v) methanol:water:chloroform as described previously (Kim et al., 2013). Ribitol (60 μl, 0.2 mg/ml, J. T. Baker, USA) was used an as internal standard (IS). Extraction was performed at 37°C with a mixing frequency of 1,200 rpm for 30 min, using a thermomixer compact (Eppendorf AG, Germany). The solutions were then centrifuged at 16,000 × *g* for 3 min. The polar phase (0.8 ml) was transferred into a new tube, and 0.4 ml of water was added. The mixed contents of the tube were centrifuged at 16,000 × *g* for 3 min. The methanol/water phase was dried in a centrifugal concentrator (CVE-2000, Eyela, Japan) for 2 h, followed by a drying process in a freeze dryer for 16 h. Methoxime (MO)-derivatization was performed by adding 80 μl of methoxyamine hydrochloride (20 mg/ml, Thermo, USA) in pyridine and shaking at 30°C for 90 min. Trimethylsilyl (TMS) etherification was performed by adding 80 μl of MSTFA (*N*-methyl-*N*-trimethylsilyltrifluoroacetamide, Sigma, St. Louis, MO, USA) at 37°C for 30 min. Gas chromatography-time of flight mass spectrometry (GC-TOFMS) was performed using a 7890A gas chromatograph (Agilent, Atlanta, GA, USA) coupled to a Pegasus HT TOF mass spectrometer (LECO, St. Joseph, MI, USA). A derivatized sample (1 μl) was separated on a 30 m × 0.25 mm I.D. fused-silica capillary column coated with 0.25 μm CP SIL 8 CB low bleed (Varian Inc., Palo Alto, CA, USA). The helium gas flow rate through the column was 1.0 ml/min. The injector temperature was 230°C, and the split ratio was set at 1:25. The temperature program was as follows: initial temperature 80°C for 2 min, followed by an increase to 320°C at 15°C/min, and a 10 min hold at 320°C. The transfer line and ion-source temperatures were 250°C and 200°C, respectively. The scanned mass range was 85–600 *m/z*, and the detector voltage was set at 1700 V.

For extraction of lipophilic metabolites, the powdered fruit samples (50 mg) were mixed with 3 ml of ethanol containing 0.1% ascorbic acid (w/v) as previously described (Kim J.K. et al., 2012). 5α-cholestane (50 μl, 10 μg/ml) was used an as IS, mixed by vortexing for 20 s, and placed in a water bath at 85°C for 5 min. After removal from the water bath, 120 μl of potassium hydroxide (80%, w/v) was added, and the samples were vortexed for 20 s and returned to the water bath for 10 min. After saponification, samples were immediately placed on ice, and deionized water (1.5 ml) was added. Hexane (1.5 ml) was then added to each sample and vortexed for 20 s, followed by centrifugation (1,200 × *g*, 5 min). The upper layer was pipetted into a separate tube, and the pellet was re-extracted using hexane. The hexane fraction was dried in a centrifugal concentrator (CVE-2000; Eyela, Tokyo, Japan). For derivatization, 30 μl of MSTFA with 30 μl of pyridine was added and incubated at 60°C for 30 min at a mixing frequency of 1,200 rpm using a thermomixer comfort (model 5355; Eppendorf AG, Hamburg, Germany).

For analyzing the lipophilic metabolites same instrument and column was used with injector temperature set to 290°C, split ratio set at 1:10, and the helium gas flow rate through the column was 1.0 ml/min. The temperature program was as follows: initial temperature 250°C, followed by an increase to 290°C at 10°C/min, and a 20 min hold at 290°C. The transfer line and the ion-source temperatures were 280 and 230°C, respectively. The scanned mass range was 50–600 *m/z*, and the detector voltage was set at 1700 V.

All analyses were performed, at least, in triplicate. Experimental data were analyzed by an analysis of variance (ANOVA) using SAS 9.2 (SAS Institute, Cary, NC, USA), and the significant differences among means were determined by Duncan’s multiple-range test. ChromaTOF software (version 4.34; LECO, St. Joseph, MI, USA) was used to support peak findings prior to quantitative analysis and for automated deconvolution of reference mass spectra. The NIST (version 2.1.2.0, Gaithersburg, MD, USA) and in-house libraries for standard chemicals were utilized for identification of the compounds. Quantitative calculation for all analytes concentrations were based on the peak area ratios for each relative to the peak area of the IS. The relative quantification data acquired from GC-TOF MS were subjected to Principal Component Analysis (PCA, SIMCA-P version 13.0; Umetrics, Umeå, Sweden) to evaluate relationships in terms of similarity or dissimilarity between groups of multivariate data. The PCA output consisted of score plots that were used for visualizing the contrasts between different samples and loading plots to explain the cluster separation. The data file was scaled with unit variance scaling before all variables were subjected to the PCA.

## Results

### Antioxidant Activity Assay of *Panax ginseng* Tissues

**FIGURE 1:**
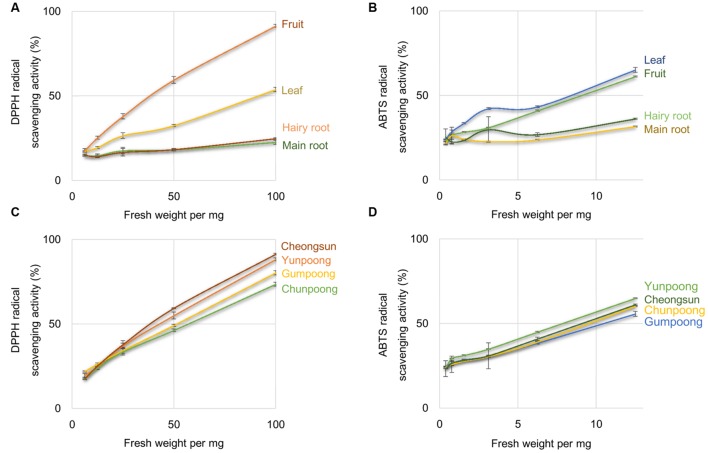
**Percentage of radical scavenging activity in different tissues and cultivars of ginseng.** Percentage of radical scavenging activity in different parts of ginseng, measured by DPPH **(A)** and ABTS **(B)** assays. Measurement of radical scavenging activity in the fruits of four cultivars of ginseng by DPPH **(C)** and ABTS **(D)** assays. Ascorbic acid was used as a positive control.

1,1-Diphenyl-2-picrylhydrazyl and ABTS radical scavenging activities assays were performed to compare the antioxidant activities of the leaves, roots, and fruits of ginseng, from four cultivars. Ascorbic acid, a well-known antioxidant, was used as a positive control. As per the DPPH assay, fruits showed maximum antioxidant activity, which was 1.7-, 3.7-, and 4.0-fold higher than leaves, hairy roots and main roots, respectively (**Figure 1A**). However, ABTS assay showed maximum antioxidant activity in the leaves followed by fruits, hairy roots, and main roots (**Figure 1B**). Out of the four ginseng cultivars, Cheongsun showed maximum antioxidant activity, followed by Yunpoong, Gumpoong, and Chunpoong as observed by the DPPH assay (**Figure 1C**). However, ABTS assay revealed highest antioxidant activity for Yunpoong followed by Cheongsun, Chunpoong, and Gumpoong (**Figure 1D**). The IC_50_ values were calculated for each sample and represented as the ratio of the IC_50_ value of the sample and IC_50_ value of ascorbic acid (positive control). The IC_50_ values of DPPH assay were 48.2 ± 1.8, 54.0 ± 2.1, 60.9 ± 2.2, and 45.8 ± 1.0 in Yunpoong, Gumpoong, Chunpoong, and Cheongsun, respectively. In the ABTS assay, IC_50_ values were 7.8 ± 0.2, 10.4 ± 0.3, 9.4 ± 0.6, and 9.0 ± 0.1, respectively (**Table 1**). Ginseng fruits showed relatively higher values of antioxidant activity compared with other tissues. Among the fruits, Cheongsun and Yunpoong showed comparatively high antioxidant activities.

**Table 1:** IC_50_ values for DPPH and ABTS radical scavenging of ginseng fruits.

	IC_50_ value ± SD	
	DPPH assay	ABTS assay
Yunpoong	48.2 ± 1.8^a^	7.8 ± 0.2^d^
Gumpoong	54.0 ± 2.1^b^	10.4 ± 0.3^e^
Chunpoong	60.9 ± 2.2^c^	9.4 ± 0.6^f^
Cheongsun	45.8 ± 1.0^a^	9.0 ± 0.1^f^

*Values with different letters indicate significant differences as observed by one-way ANOVA followed by Tukey’s Kramer *post hoc* test (*p* < 0.05).*

### Proteome Analysis of Ginseng Fruits (cv. Cheongsun)

**FIGURE 2:**
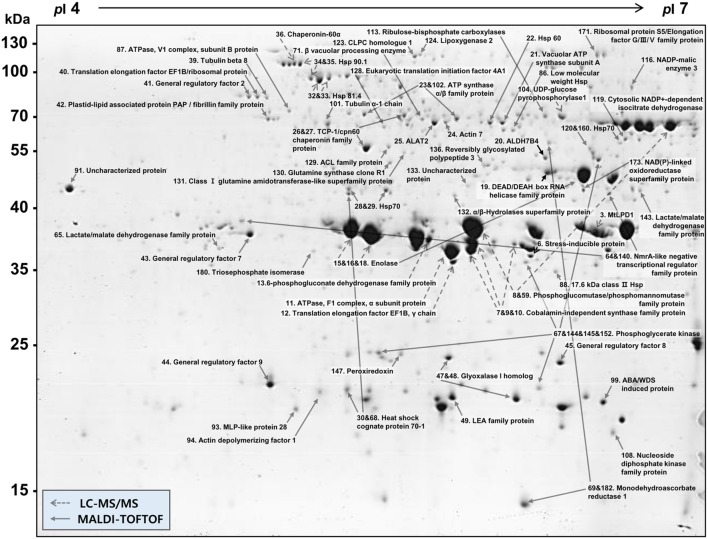
**2D gel reference map of the ginseng fruit (cv. Cheongsun).** This map represents the protein information of 81 spots identified by MALDI-TOF/TOF MS and/or LC-MS/MS.

**FIGURE 3:**
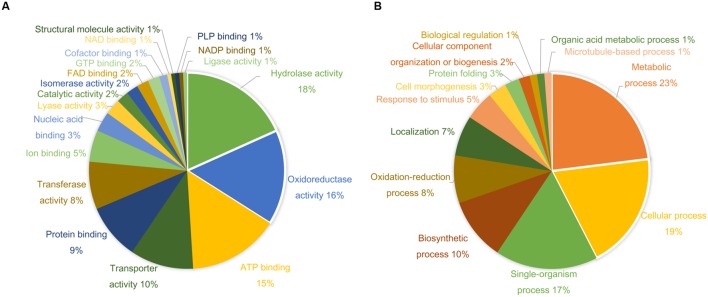
**Gene ontology (GO) analysis shows diverse functional categories of the identified proteins.** The percentage distribution of reported GO terms is shown in the two pie charts. **(A)** Proteins involved in molecular function categories. **(B)** Proteins involved in biological process categories.

More than 400 spots were detected in the 2D maps of ginseng fruit proteome. Of which, 182 well-separated spots were subjected to MALDI-TOF/TOF MS or/and LC-MS/MS resulting in successful identification and protein assignment of 81 spots with high-confidence (Supplementary Tables 1 and 2). These identifications allowed to develop a 2D reference map for the Cheongsun cultivar (**Figure 2**) and submitted to World-2DPAGE repository (database no. 85). All information regarding the 2D map and identified proteins can be accessed online using following link^1^. As the genome of the ginseng is not sequenced and MS/MS spectra were searched in an in-house developed RNAseq database (srx1791675), a manual accession number was given to all the identified proteins. Gene ontology analysis showed that the identified proteins were mainly associated with the hydrolase (18%), oxidoreductase (16%), and ATP binding (15%) activities (**Figure 3**). In biological process category, proteins related to metabolic (23%), cellular (19%), and single-organism (17%) processes were identified.

**FIGURE 4:**
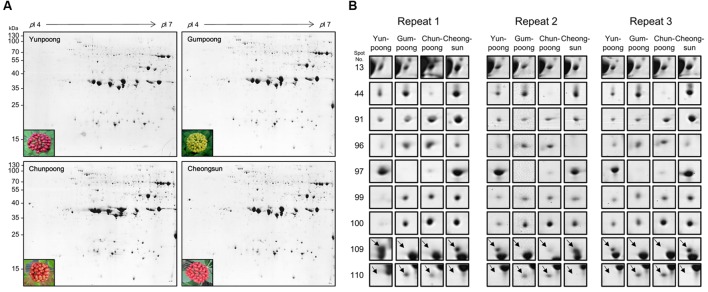
**Comparative proteome analysis of four cultivars ginseng fruits. (A)** Representative 2D gel profiles and pictures of four cultivars of ginseng fruits. Yunpoong and Cheongsun have red berries, Gumpoong has yellow berries and Chunpoong has orange berries. **(B)** Enlarged views of differentially modulated proteins in these four cultivars.

A comparative analysis of four ginseng cultivars was also carried out to gain insight into the cultivar-specific fruit proteins (**Figures 4A,B**). A total of 27 differentially modulated protein spots were observed among the four cultivars, and 22 proteins were identified. Gene ontology analysis showed these differentially expressed proteins were mainly associated with the ATP binding (28%), protein binding (20%), and oxidoreductase activity (12%). In the biological process category, proteins related to metabolic (19%), cellular (19%), and response to stimulus (14%) processes were identified.

### Metabolome Analysis of the Ginseng Fruits

**FIGURE 5:**
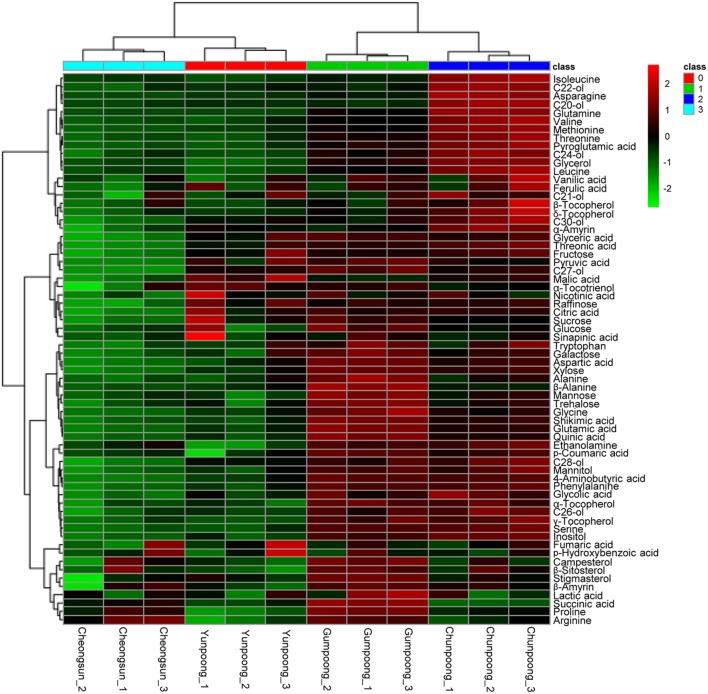
**GC-TOF MS analysis of the metabolites from four cultivars of ginseng fruits.** Heat map shows the metabolite profiles of the fruits. Red and green colors indicate that the metabolite content is increased and decreased, respectively. C20-ol: eicosanol, C21-ol: heneicosanol, C22-ol: docosanol, C24-ol: tetracosanol, C26-ol: hexacosanol, C27-ol: heptacosanol, C28-ol: octacosanol, C30-ol: triacontanol.

Metabolome analysis of four cultivars of ginseng fruits was carried out using GC-TOF MS. Metabolite profiles were presented as the heat map, PCA score plots, and PCA loading plots. A total of 66 metabolites were identified and quantified, wherein 48 were hydrophilic and 18 were lipophilic (Supplementary Tables 3 and 4). Based on these identifications, metabolite information was submitted to MetaboLights public repository (database no. MTBLS350). All information regarding the identified metabolites can be accessed online using following link^2^. The content of lipophilic metabolites in ginseng fruits was indicated in **Table 2**. Heat map clearly showed higher amounts of metabolites in Gumpoong and Chunpoong cultivars as compared to Cheongsun and Yunpoong (**Figure 5**). The contents of nine amino acids (alanine, proline, glycine, β-alanine, aspartic acid, 4-aminobutyric acid, arginine, glutamic acid, and tryptophan), seven sugars (xylose, galactose, glucose, mannose, mannitol, sucrose, and trehalose), six organic acids (pyruvic acid, lactic acid, succinic acid, shikimic acid, quinic acid, and *p-*coumaric acid), six organic compounds (C27-ol, α-tocopherol, campesterol, stigmasterol, β-sitosterol, and β-amyrin), and sugar acids (glyceric acid) were higher in Gumpoong, whereas fourteen organic compounds (ethanolamine, glycerol, inositol, C20-ol, C21-ol, C22-ol, C24-ol, C26-ol, C28-ol, C30-ol, β-tocopherol, δ-tocopherol, γ-tocopherol, and α-amyrin), 10 amino acids (valine, leucine, isoleucine, serine, threonine, methionine, pyroglutamic acid, phenylalanine, asparagine, and glutamine), two organic acids (glycolic acid and ferulic acid), two sugars (fructose and mannitol), a sugar acid (threonic acid), and a phenolic acid (vanillic acid) were enriched in Chunpoong.

**Table 2:** Content (μg/g of dry weight) of lipophilic metabolites in ginseng fruits.

	Yunpoong	Gumpoong	Chunpoong	Cheongsun
C20-ol	7.17 ± 0.19^a^	7.27 ± 0.91^a^	27.70 ± 1.33^b^	4.96 ± 1.18^a^
C21-ol	2.58 ± 0.34^a^	2.56 ± 0.22^a^	2.88 ± 0.21^a^	2.30 ± 0.44^a^
C22-ol	3.49 ± 0.27^a^	3.60 ± 0.24^a^	6.27 ± 0.12^b^	2.86 ± 0.51^a^
C24-ol	1.53 ± 0.05^a^	2.18 ± 0.10^b^	2.87 ± 0.10^c^	1.51 ± 0.28^a^
C26-ol	2.05 ± 0.07^a^	2.61 ± 0.15^b^	2.79 ± 0.15^b^	1.85 ± 0.25^a^
C27-ol	0.67 ± 0.03^a^	0.91 ± 0.10^b^	0.69 ± 0.08^a^	0.00^c^
C28-ol	3.80 ± 0.06^a^	4.72 ± 0.39^ab^	5.23 ± 0.55^b^	2.16 ± 0.40^c^
C30-ol	14.35 ± 1.07^a^	14.81 ± 1.99^a^	21.41 ± 2.57^b^	8.25 ± 1.62^c^
α-Tocopherol	181.42 ± 29.17^a^	293.74 ± 12.46^b^	278.58 ± 17.62^b^	164.58 ± 32.64^c^
β-Tocopherol	6.41 ± 0.65^a^	7.63 ± 1.25^a^	10.17 ± 2.01^a^	6.49 ± 1.85^a^
γ-Tocopherol	10.38 ± 0.24^a^	24.30 ± 2.75^b^	25.47 ± 3.10^b^	10.05 ± 3.02^a^
δ-Tocopherol	3.30 ± 0.16^a^	4.28 ± 0.56^a^	6.68 ± 1.13^b^	2.77 ± 0.89^a^
α-Tocotrienol	26.81 ± 2.02^a^	25.41 ± 0.71^a^	22.65 ± 0.81^a^	18.35 ± 6.39^a^
α-Amyrin	0.59 ± 0.03^a^	0.56 ± 0.04^a^	0.81 ± 0.04^b^	0.38 ± 0.11^c^
β-Amyrin	16.13 ± 1.23^a^	20.19 ± 0.41^a^	18.50 ± 0.72^a^	16.64 ± 4.36^a^
Campesterol	43.82 ± 1.21^a^	49.77 ± 0.53^a^	45.33 ± 1.99^a^	45.65 ± 4.25^a^
β-Sitosterol	620.32 ± 33.12^a^	777.81 ± 13.40^b^	722.00 ± 59.14^b^	701.83 ± 95.74^b^
Stigmasterol	215.67 ± 13.85^a^	255.11 ± 1.03^ab^	219.11 ± 10.18^a^	190.16 ± 38.84^ac^

*Each value represents the mean ± standard deviation (*n* = 3). Values with different letters indicate significant differences as observed by one-way ANOVA followed by Tukey’s Kramer *post hoc* test (*p* < 0.05). C20-ol, eicosanol; C21-ol, heneicosanol; C22-ol, docosanol; C24-ol, tetracosanol; C26-ol, hexacosanol; C27-ol, heptacosanol; C28-ol, octacosanol; C30-ol, triacontanol.*

**FIGURE 6:**
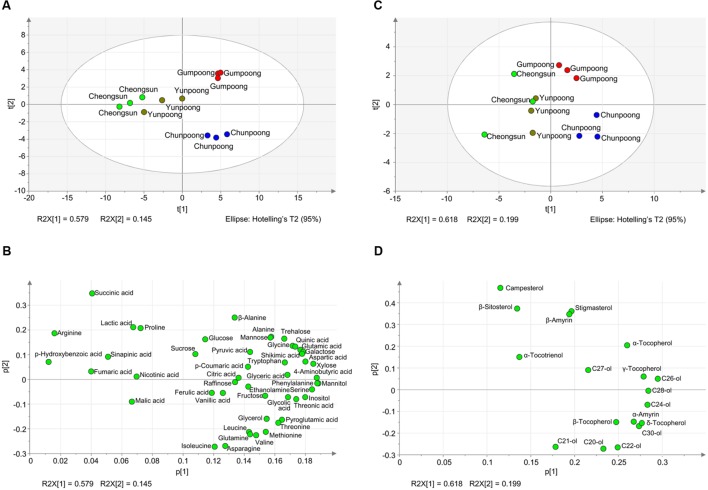
**Metabolite profiles of the four ginseng fruits presented by PCA score plots and PCA loading plots. (A)** PCA score plot for hydrophilic metabolites profiling of the four cultivars. The ellipse indicates the 95% confidence border based on Hotelling’s T^2^. **(B)** PCA loading plot for the hydrophilic metabolites. The corresponding PCA loading plot showing metabolites that contribute to the separation observed on the score plot. **(C)** PCA score plot for lipophilic metabolites profiling. **(D)** PCA loading plot for the lipophilic metabolites. C20-ol, eicosanol; C21-ol, heneicosanol; C22-ol, docosanol; C24-ol, tetracosanol; C26-ol, hexacosanol; C27-ol, heptacosanol; C28-ol, octacosanol; C30-ol, triacontanol.

To further depict the distribution of metabolites among each cultivar and to find out the similarity and differences between metabolite profiles among the four cultivars, PCA analysis was carried out. The PCA loading plot showed the metabolites that contributed to the separation on PC1 and PC2 (**Figure 6**). Gumpoong and Chunpoong were separated from Yunpoong and Cheongsun at the PC2 axis while Gumpoong and Chunpoong were separated from each other at PC1 axis (**Figure 6A**). Along PC1, xylose, phenyl alanine, mannitol, and serine were observed as major contributors to the discrimination of Gumpoong and Chunpoong cultivars. Along PC2, a high level of succinic acid was detected in Gumpoong, whereas isoleucine and asparagine were abundant in Chunpoong (**Figure 6B**). Separation pattern of the lipophilic metabolites in the PCA score plot was also similar with that of the hydrophilic metabolites (**Figure 6C**). C24-ol, C26-ol, and C28-ol were detected as major contributors to the discrimination of Gumpoong and Chunpoong. A high level of campesterol was detected in Gumpoong, whereas C20-ol, C21-ol, and C22-ol were abundant in Chunpoong (**Figure 6D**).

## Discussion

### Ginseng Fruits Have Higher Antioxidant Activity

1,1-Diphenyl-2-picrylhydrazyl and ABTS assays were performed to confirm the antioxidant activity in different tissues of ginseng, where fruits showed higher antioxidant activities compared to other tissues (**Figure 1**). In fruit, Cheongsun and Yunpoong showed comparatively higher antioxidant activities over the other two cultivars. DPPH assay showed higher level of antioxidant activities than ABTS assay. ABTS assay is based on the detection of blue/green ABTS^+^, which can measure both hydrophilic and lipophilic antioxidants, whereas DPPH assay is appropriate only for the detection of the hydrophobic antioxidants. Ginseng fruits are rich in ginsenosides (Ko et al., 2008; Kim et al., 2009) and it was reported that ginsenosides increase antioxidant enzyme activity and function as free-radical scavengers (Lu et al., 2009). Total ginsenosides of ginseng fruits were reported to be four times higher than those of 4-year-old ginseng roots (Ko et al., 2008). Yunpoong cultivars showed greater amount of ginsenosides than Gumpoong and Chunpoong cultivars (Kim et al., 2009). In light of these findings, it can be concluded that the high level of antioxidant activity in ginseng fruits may be partially related to the ginsenosides.

### 2D Gel Reference Map of Ginseng Fruit Proteome

As of today, no report has been published on the proteomic analysis of ginseng fruits. Therefore, here we investigated the ginseng fruit proteome using high-resolution 2-DE. Out of 182 well separated spots subjected to the MS analysis, only 24 protein spots were identified (about 13% of total), when searched in the NCBInr and UniRef databases. One of the reasons for this comparatively lesser identification is the lack of a ginseng genome database. Therefore, to overcome this bottleneck and to increase the protein identification, we developed an in-house RNAseq database with information of 59,251 proteins. After searching the MS/MS spectra against this RNAseq database, protein identification ratio was increased to almost 45%. Hence, the total number of identified protein spots increased to 81.

The majority of the identified proteins were associated with the hydrolase activity, oxidoreductase activity, and metabolic processes. During ripening, fruits enlarge by cell expansion, which requires cell wall elongation and accumulation of solutes within the vacuole. Therefore, identified proteins related to cell cytoskeleton, senescence, maturation, and differentiation like DEAD/DEAH box RNA helicase (spot 19), actin (spot 24), tubulin (spot 39, 101), plastid-lipid associated protein (PAP)/fibrillin (spot 42), vacuolar processing enzyme (spot 71), major latex protein (MLP)-like protein (spot 93), and actin depolymerizing factor 1 (spot 94), were identified. Glycolysis and tricarboxylic acid (TCA) cycle are the major energy sources for the cells and thus proteins related to these processes are expected to provide necessary energy required for the fruit ripening or cell expansion. Some of these proteins were identified here and included enolase (spot 15, 16, 18), triosephosphate isomerase (spot 59, 180), phosphoglycerate kinase (spot 67, 144, 145, 152), ATPase (spot 87), nucleoside diphosphate kinase family protein (spot 108), ribulose-bisphosphate carboxylase (spot 113), hydrolase (spot 132), and NAD(P)-linked oxidoreductase (spot 173).

Fruit ripening is an oxidative process where H_2_O_2_ and reactive oxygen species (ROS) accumulation are balanced by antioxidant systems (Jimenez et al., 2002). Therefore, several antioxidant proteins could be identified in the ginseng fruits. Aldehyde dehydrogenase 7B4 (spot 20) plays a major role in the detoxification processes of aldehydes, generated in plants, when exposed to abiotic stress. It functions as an aldehyde-detoxifying enzyme, which is an efficient ROS scavenger, and also acts as lipid peroxidation-inhibiting enzyme (Kotchoni et al., 2006). Monodehydroascorbate reductase 1 (spot 69) catalyzes the generation of ascorbate and NAD^+^ from NADH and H^+^. It is an enzymatic component of the glutathione-ascorbate cycle which is one of the major antioxidant machineries in the plant cells (Leterrier et al., 2005). Peroxiredoxin (spot 147) is family of antioxidant enzymes that also control cytokine-induced peroxide levels and thereby mediate signal transduction in mammalian cells. It is related to the cell differentiation and proliferation during apoptosis, which is linked to the antioxidative activities (Rouhier and Jacquot, 2002). Glyoxalase 1 homolog (spot 47) is part of the glyoxalase system which detoxifies methylglyoxal and the other reactive aldehydes present in cell cytosol. Methylglyoxal is a reduced derivative of pyruvic acid and formed as a side-product of several metabolic pathways (Thornalley, 2003). Although, there is no direct involvement of glyoxalase 1 in ROS detoxification, it regulates the ROS by controlling methylglyoxal concentrations (Gupta and Deswal, 2012). High concentration of methylglyoxal inhibits glutathione peroxidase activity that leads to ROS accumulation (Park et al., 2003). On the contrary, glyoxalase 1 reduces the levels of methylglyoxal and thus regulates the glutathione peroxidase activity for proper ROS detoxification.

The other identified proteins included heat shock proteins (HSPs) such as the low molecular weight HSP (spot 86) and 17.6 kDa class 2 HSP (spot 88). These proteins are generally related to stress responses and expressed during tissue remodeling. The major function of these proteins is to stabilize the protein structure, some of these like small HSPs which have 15–30 kDa molecular mass, are also involved in decreasing the intracellular ROS in a glutathione-dependent way (Sun et al., 2002). Besides, three isoforms of cobalamin-independent synthase family protein (spots 7, 9, and 10) were identified, and which is involved in the methionine biosynthesis.

Based on these identifications, a 2D gel reference map for the ginseng fruit proteome was developed. 2D gel reference maps allows functional annotation of all identified proteins according to biological categories as well as display the annotation of several proteins per analyzed proteins “spot” according to MS primary data (Rode et al., 2011). Several proteome reference maps have been constructed in the last decade which serves as useful tools to identify proteins of analogous protein fractions by simple spot pattern comparison. Currently, these maps are shared online through the World-2DPAGE Repository^3^. Even though we developed an in-house RNAseq database for protein identification, these are still insufficient as compared to model crops such as rice and soybeans whose genomes have been sequenced. The absence of a ginseng reference genome database is a major bottleneck in ginseng research. However, we suggest that a reference map is much more helpful due to incomplete genomic sequencing and will serve as a resource for comparative parallel studies.

### Comparative Proteome Analysis of Fruits of Four Ginseng Cultivars

A comparative analysis of ginseng fruit proteins from four cultivars was carried out which revealed 27 differentially modulated protein spots, of which only 22 were successfully assigned for protein identity. Spot 13 was differently expressed in the four cultivars: Cheongsun showed the highest expression level followed by Chunpoong, Yunpoong, and Gumpoong. Spot 13 was identified as a 6-phosphogluconate dehydrogenase (6PGDH) family protein. This protein catalyzes the third step of the pentose phosphate pathway, which involves generation of ribulose-5-phosphate (Ru5P) and CO_2_ from 6-phosphogluconate with concomitant reduction of NADP^+^ to NADPH. This reaction is a key component of the hexose monophosphate shunt (Broedel and Wolf, 1990) and pentose phosphate pathways (PPP; Adams et al., 1983). However, very little is known on the function(s) of 6PGDH family proteins. General regulatory factor (GRF) 9 (spot 44) was highly expressed in Gumpoong and Cheongsun as compared to the other two cultivars. This protein is associated with a G box DNA/protein complex, which is a well-characterized *cis*-acting DNA regulatory element found in plant genes. This protein functions as a receptor for the phytotoxin fusicoccin in several plants (Korthout and Deboer, 1994; Oecking et al., 1994), and regulates nitrate reductase (Moorhead et al., 1996). The ABA/WDS induced protein (spot 99) was differentially expressed in all cultivars and showed highest expression level in Cheongsun, followed by Chunpoong, Gumpoong, and Yunpoong. This protein is induced by water deficit stress (WDS; Padmanabhan et al., 1997) or abscisic acid (ABA) stress and ripening (Canel et al., 1995). In another study, transgenic *Arabidopsis* plants overexpressing the *Asr* gene [ABA/WDS category (PF02496)] exhibited reduced sensitivity to ABA and levels of dormancy, whereas increased resistance to salt, osmotic, drought, and cold stresses was seen (Gonzalez and Lusem, 2014). Introduction of this protein to other plants might contribute to increased stress tolerance against salt, osmotic, drought, and cold.

### Ginseng Fruits Are Rich in Medicinal Compounds

In previous studies, primary metabolites were analyzed in the ginseng roots (Lee et al., 2009). However, there is no report on the ginseng fruit metabolome as a whole. Ginseng fruits mainly contain secondary metabolites such as saponins and phenolic compounds (Ko et al., 2008). Medicinal properties of ginseng is primarily because of the presence of ginsenosides, therefore previous efforts on ginseng fruit analysis were mainly focused on the identification and quantification of ginsenosides. Thus, the information regarding other primary and secondary metabolites of ginseng fruits was missing. Here we used GC-TOF MS for the identification of both primary and secondary metabolites of ginseng fruits. A total of 66 metabolites including amino acids, sugars, organic acids, phenolic acids, phytosterols, tocopherols, and policosanols were identified and quantified. Both *p-*hydroxybenzoic acid (PHBA) and vanillic acid are phenolic derivatives of benzoic acid and have been identified in a number of plants (Pugazhendhi et al., 2005). PHBA is well known for its medicinal effects and exhibit antifungal, antimutagenic, antisickling, antimicrobial (Chong et al., 2009), and estrogenic activities. Similarly, vanillic acid has also been shown to possess antisickling and anthelmintic activities. Moreover, it was also shown that vanillic acid suppress the hepatic fibrosis in chronic liver injury (Itoh et al., 2009). Being the phenolic derivatives of hydroxycinnamic acids, *p*-coumaric acid, sinapinic acid, and ferulic acid are known to have antioxidant, hypoglycemic, antiviral, and hepatoprotective activities (Farah and Donangelo, 2006). In addition, ferulic acid also exhibits antiallergic, hepatoprotective, anticarcinogenic, anti-inflammatory, vasodilatory, and antithrombotic effects, and helps to increase the viability of sperms (Kumar and Pruthi, 2014). Interestingly, all of these metabolites were previously identified in ginseng leaves and roots (Jung et al., 2002; Seong et al., 2005), indicating their presence throughout the ginseng plant irrespective of the tissue type. A further comparison of the identified metabolites between fruits and roots led to the identification of several fruit-specific metabolites, which were mainly policosanols, tocopherols, organic acids, and phenolic acids. Phenolic acids and organic acids, specific to fruits include nicotinic acid, quinic acid, and shikimic acid. Nicotinic acid is a water-soluble vitamin of the B complex and is the precursor of several alkaloids commonly known as the “pyridine alkaloids” which have several medicinal properties. Similarly, shikimic acid is also a precursor for the antiviral drug oseltamivir (Ghosh et al., 2012) and antibacterial compound (6S)-6-fluoroshikimic acid (Davies et al., 1994). Besides, shikimic acid is also involved in the formation of polyphenol flavonoids having antioxidant properties (Shao et al., 2007). Out of the lipophilic metabolites identified here, β-sitosterol, β-amyrin, stigmasterol, and campesterol have previously been reported in the ginseng roots while others were unique to the fruits. Tocotrienols and tocopherols have vitamin E activity and thus can prevent cellular damage from oxidative stress (Sen et al., 2006). Tocopherol is frequently used in foods as an antioxidant. Furthermore, tocopherol also slows down the progression of Alzheimer’s disease (Sano et al., 1997) and exhibits positive effects in Parkinson syndrome (Fahn, 1992). Phytosterols reduce low-density lipoprotein (LDL) cholesterol levels, and increase high-density lipoprotein (HDL) cholesterol levels in the blood (Jones et al., 1997). Policosanol is a mixture of alcohols which are mostly extracted from plant wax but may also be obtained from cereal grains, fruits, and beeswax (Marinangeli et al., 2010). Moreover, policosanol also inhibits platelet aggregation (Arruzazabala et al., 1993), prevents cardiovascular diseases (Varady et al., 2003), and effects as an alternative to aspirin in patients suffering from gastric irritation (Taylor et al., 2003).

Policosanols, tocopherols, triterpenes, and phytosterols have previously been reported in the other medicinal fruits. However, the contents of most of these metabolites were found to be higher in ginseng fruits as compared to the other fruits reported previously. The α-tocopherol, β-tocopherol, γ-tocopherol, β-amyrin, β-sitosterol, and stigmasterol were 48.3%, 498.3%, 7.7%, 56.4%, 101.1%, and 326.4% higher in ginseng fruits compared to the fruits of wolfberry (*Lycium chinense* Mill., Park et al., 2012). Moreover, the amounts of campesterol, stigmasterol, and β-sitosterol were 250.5%, 452.2%, and 652.2% higher in ginseng than elderberry (*Sambucus nigra* L.; Salvador et al., 2015). In contrast, α-amyrin and campesterol was lower in ginseng than wolfberry and C26-ol and β-amyrin were lower than elderberry. In comparison with the Indian ginseng (*Withania somnifera* L.), α-tocopherol, campesterol, β-sitosterol, and stigmasterol were found to be 4779.4, 202.7, 6063.3, and 1924.7% higher in ginseng fruits; however, the content of β-tocopherol was lower (Bhatia et al., 2013).

### Comparative Metabolome Analysis of Ginseng Fruits Revealed Higher Abundance of Metabolites in Chunpoong and Gumpoong Cultivars

A comparative analysis of the fruit metabolites showed that Chunpoong had higher amounts of medicinal compounds than other three cultivars. Isoleucine, C22-ol, asparagine, C20-ol, and glutamine were highly enriched in Chunpoong, whereas β-sitosterol, stigmasterol, campesterol, β-amyrin, and glucose were highly detected in Gumpoong and a relatively high amount of α-tocotrienol and malic acid were detected in Yunpoong. Arginine which is highly enriched in Gumpoong is a substrate for nitric oxide synthase that generate nitric oxide (NO). NO has been associated with the putative antioxidant and anticancer properties of ginseng (Friedl et al., 2001). These compounds could potentially serve as biomarkers to distinguish between ginseng cultivars. Analysis of ginseng metabolites enables understanding and regulation of its associated medicinal properties, which could also be useful in developing commercial products. Metabolites can play a role as biomarkers for disease diagnosis (An et al., 2005) and drug development and alter detection associated with plant development (Tarpley et al., 2005). These results also suggest that two cultivars (Gumpoong and Chunpoong) with higher amounts of metabolites might have high levels of biological activity and possess beneficial substances for health.

## Conclusion

Previous research showed that saponin content of the ginseng fruits is higher than of roots, indicating that ginseng fruits may also exhibit medicinal properties similar to the roots. However, and to the best of our knowledge, no research is available in support of the medicinal effects of ginseng fruits. Our experimental approach was targeted toward identifying the molecular components within ginseng fruits in support of the hypothesis that these fruits exhibit medicinal value. We first compared the antioxidant activities in different ginseng tissues, results of which showed that ginseng fruits exhibit higher antioxidant activities than rest of the tissues. As a next step, the proteome of ginseng fruits was analyzed to identify any pathway(s) associated with the production of medicinal compounds; however, due to limitations in protein identification no associated pathway/s could be discerned. Nevertheless, we were able to identify 81 proteins from ginseng fruits associated with the hydrolase, oxidoreductase, and ATP binding activities. Oxidoreductases are involved in the redox regulation and thus contribute to the antioxidant activities. Therefore, identification of these enzymes in ginseng fruits supports the higher antioxidant activities of ginseng fruits. Next, we analyzed the metabolome of ginseng fruits. As the medicinal compounds are generally volatile in nature, we employed a GC-TOF MS-based analysis revealing that ginseng fruits are rich in some of the well-known medicinal compounds. Further, the concentration of most of these metabolites including α-tocopherol, β-tocopherol, γ-tocopherol, β-amyrin, β-sitosterol, and stigmasterol, among others, is higher in the ginseng fruits as compared to the other plants and ginseng roots. Interestingly, we identified some novel metabolites including policosanols, tocopherols, organic acids, and phenolic acids, which have not yet been reported in the ginseng plant. These results show that ginseng fruits are rich in medicinal compounds. Finally, comparing the four ginseng cultivars to find out which cultivar is richer in the medicinal compounds, we identified Chunpoong and Gumpoong cultivars as having higher amounts of the medicinal metabolites. In all, we believe that a deeper understanding of the molecular components of ginseng fruits, particularly related to their medicinal value would help in the further study of the underutilized part of the ginseng plant with potential health-related benefits.

## Accession Codes

2D reference map with mass spectrometry proteomic data were deposited in the World-2DPAGE repository^4^; database number 0085. Metabolites data were deposited in the MetaboLights^5^; database number MTBLS350.

## Conflict of Interest Statement

The authors declare that the research was conducted in the absence of any commercial or financial relationships that could be construed as a potential conflict of interest.

## Footnotes

1 http://world-2dpage.expasy.org/repository/database=0085

2 http://www.ebi.ac.uk/metabolights/MTBLS350

3 http://world-2dpage.expasy.org/repository/

4 http://world-2dpage.expasy.org

5 http://www.ebi.ac.uk/metabolights/
